# Rab11a Controls Cell Shape via C9orf72 Protein: Possible Relationships to Frontotemporal Dementia/Amyotrophic Lateral Sclerosis (FTDALS) Type 1

**DOI:** 10.3390/pathophysiology31010008

**Published:** 2024-02-09

**Authors:** Shoya Fukatsu, Hinami Sashi, Remina Shirai, Norio Takagi, Hiroaki Oizumi, Masahiro Yamamoto, Katsuya Ohbuchi, Yuki Miyamoto, Junji Yamauchi

**Affiliations:** 1 Laboratory of Molecular Neurology, Tokyo University of Pharmacy and Life Sciences, Tokyo 192-0392, Japanrshirai@toyaku.ac.jp (R.S.); miyamoto-y@ncchd.go.jp (Y.M.); 2 Laboratory of Applied Biochemistry, Tokyo University of Pharmacy and Life Sciences, Tokyo 192-0392, Japan; y23301@toyaku.ac.jp (H.S.); takagino@toyaku.ac.jp (N.T.); 3 Tsumura Research Laboratories, Tsumura & Co., Inashiki 200-1192, Japan; ooizumi_hiroaki@mail.tsumura.co.jp (H.O.);; 4 Laboratory of Molecular Pharmacology, National Research Institute for Child Health and Development, Tokyo 157-8535, Japan; 5 Diabetic Neuropathy Project, Tokyo Metropolitan Institute of Medical Science, Tokyo 156-8506, Japan

**Keywords:** Rab, FTDALS, hesperetin, N1E-115, FBD-102b

## Abstract

Abnormal nucleotide insertions of C9orf72, which forms a complex with Smith–Magenis syndrome chromosomal region candidate gene 8 (SMCR8) protein and WD repeat-containing protein 41 (WDR41) protein, are associated with an autosomal-dominant neurodegenerative frontotemporal dementia and/or amyotrophic lateral sclerosis type 1 (FTDALS1). The differentially expressed in normal and neoplastic cells (DENN) domain-containing C9orf72 and its complex with SMCR8 and WDR41 function as a guanine-nucleotide exchange factor for Rab GTP/GDP-binding proteins (Rab GEF, also called Rab activator). Among Rab proteins serving as major effectors, there exists Rab11a. However, it remains to be established which Rab protein is related to promoting or sustaining neuronal morphogenesis or homeostasis. In this study, we describe that the knockdown of Rab11a decreases the expression levels of neuronal differentiation marker proteins, as well as the elongation of neurite-like processes, using N1E-115 cells, a well-utilized neuronal differentiation model. Similar results were obtained in primary cortical neurons. In contrast, the knockdown of Rab11b, a Rab11a homolog, did not significantly affect their cell morphological changes. It is of note that treatment with hesperetin, a citrus flavonoid (also known as Vitamin P), recovered the neuronal morphological phenotypes induced by Rab11a knockdown. Also, the knockdown of Rab11a or Rab11b led to a decrease in glial marker expression levels and in morphological changes in FBD-102b cells, which serve as the oligodendroglial differentiation model. Rab11a is specifically involved in the regulation of neuronal morphological differentiation. The knockdown effect mimicking the loss of function of C9orf72 is reversed by treatment with hesperetin. These findings may reveal a clue for identifying one of the potential molecular and cellular phenotypes underlying FTDALS1.

## 1. Introduction

In the development of the central nervous system (CNS), neuronal and glial cells independently or cooperatively undergo continuous and dynamic morphological changes [1,2,3,4,5,6]. In neuronal cells, morphological changes are involved in a variety of stages. In the beginning in embryos, neurites outgrow and elongate [1,2,3,4,5,6]. The long neuronal processes navigate to the final points where synapses are transiently formed [3,4,5,6]. The neural networks eventually become configured to accommodate plasticity, together with the interaction with glial cells [3,4,5,6]. The molecular mechanisms underlying their cell morphogenesis, however, remain to be understood.

On the other hand, in neurological diseases, especially in neurodegenerative disorders, neuronal and glial morphogenesis can be affected not only in the embryonic stage but also in the adult stage. Abnormal morphogenesis in neuronal and glial cells is associated with the onset or the specific phenotype of neurological disorders [7,8,9,10,11,12]. Frontotemporal dementia (FTD) and/or amyotrophic lateral sclerosis (ALS) is one such disease that is a genetically heterogeneous autosomal-dominant neurodegenerative disorder [7,8,9,10,11,12]. FTD/ALS is considered to be a single-spectrum disorder that is caused by various types of mutations [7,8,9,10,11,12].

FTDALS-associated gene products including FTDALS1 likely converge on some common pathological molecular pathways [13,14]. This common disease-related pathway may explain the FTD and ALS overlap in clinical symptoms [13,14]. One of the major molecular pathological mechanisms of FTDALS appears to include abnormality or deficiency of proteostasis [13,14]. For example, there may be unsuitable timing of protein degradation and its decreased or increased amount [13,14]. The networks of proteostasis include integrated cellular biological pathways in living cells and control the biosynthesis, folding, transport, and degradation of a variety of proteins on the inside, surface, and outside of cells [15,16]. Sustaining these protein metabolic pathways is homeostatically associated with cell morphological changes and differentiation [15,16]. Additionally, the products of the gene responsible for FTDALS directly or indirectly control intracellular small GTPases, which are the central regulators of morphological changes and tissue morphogenesis as well as biological pathways composing proteostasis.

The mean age at the onset for FTDALS1 patients is 40 to 45 years. Almost all patients exhibit cognitive impairment, executive dysfunction, and/or memory problems, accompanied by psychiatric and/or behavioral problems. Additional prominent features involve movement disorders such as chorea, dystonia, myoclonus, tremor, and rigidity [7,8,9,10,11,12]. In many phenotypic categories, these phenotypes distinguish FTDALS1 from the cases of other FTDALSs. FTDALS1 is caused by a hexanucleotide (GGGGCC) repeat expansion in a noncoding region of the *c9orf72* gene [7,8,9,10,11,12]. Its insertion results in the loss of function of the C9orf72 protein [7,8,9,10,11,12,13,14]. In addition, the presence of toxic dipeptide repeat proteins generated by nucleotide repeat-associated non-ATG translation is known [7,8,9,10,11,12,13,14]. While dipeptide repeat proteins exert multiple toxic effects on cells, the precise connection between the functional loss of C9orf72 and undifferentiated, as well as subsequent degradative, phenotypes in neuronal cells remains to be determined.

Among the Rab proteins mediating proteostasis as major effectors, there exists Rab11a, which has multiple functions, including intracellular vesicle trafficking [17,18,19,20]. However, it remains to be established which and how Rab protein, acting downstream of C9orf72, is primarily related to affecting neuronal and glial morphological changes or homeostasis. Like other Rab proteins, Rab11a and its homolog Rab11b are important regulators of membrane trafficking within cells [17,18,19,20]. Rab11a and Rab11b act as key regulators around the plasma membrane and perinuclear region [17,18,19,20], and are also localized to early endosomes and recycling endosomes. They are thought to be involved in the transport from late endosomes to lysosomes [17,18,19,20]. In particular, Rab11a plays a central role in the homeostasis of endosome–lysosome biogenesis. Since biological pathways composing proteostasis centered on the endosome–lysosome system are associated with cell morphological changes and differentiation [15,16], it is presumed that Rab11a and/or Rab11b participate in morphological differentiation in neuronal cells.

Herein, we describe that the knockdown of Rab11a but not Rab11b decreases the expression levels of neuronal differentiation marker proteins as well as morphological changes in N1E-115 cells, which serve as a widely used model cell undergoing neuronal differentiation [21,22]. Similar results were obtained in primary cortical neurons. Their decreased morphological changes by Rab11a knockdown are recovered by treatment with hesperetin, a flavonoid with neuronal-protective effects [23,24,25,26]. These effects are supported by the results of phosphorylation states of mitogen-activated protein kinase/extracellular signal-regulated protein kinase (MAPK/ERK) [27,28,29]. Also, the knockdown of Rab11a or Rab11b affects morphological changes using FBD-102b cells as oligodendroglial differentiation model cells [29,30]. These studies are expected to contribute to our understanding of the potential molecular and cellular pathological mechanisms underlying FTDALSs and the functional deficiency of Rab protein.

## 2. Materials and Methods

### 2.1. Antibodies and Chemicals

The key materials including antibodies used in this experiment are listed in Table 1.

### 2.2. Synthetic siRNAs and DNA Primers

The 19-mer short interfering (si)RNAs with tandem deoxythymidine dinucleotides (dTdT) (Fasmac, Atsugi-shi, Japan) and DNA primers (Fasmac) are described in the supplemental figures. The control siRNA sequence is described in Ref. [29].

### 2.3. Reverse Transcription-Polymerase Chain (RT-PCR) Reaction

The cDNAs were prepared from the total RNA extracted using Isogen (Nippon Gene, Tokyo, Japan) with the PrimeScript RT Master Mix kit (Takara Bio, Kyoto, Japan) in accordance with the manufacturer’s instructions. PCR amplification from reverse transcription products was performed using Gflex DNA polymerase (Takara Bio) with 30 to 36 cycles, each consisting of a denaturation reaction at 98 °C (0.2 min), an annealing reaction at 56 to 65 °C (0.25 min) depending on the annealing temperature, and an extension reaction at 68 °C (0.5 min). The resultant PCR products were loaded onto 1% to 2% of premade agarose gels (Nacalai Tesque, Kyoto, Japan, or Fujifilm, Tokyo, Japan).

### 2.4. Cell Culture and Differentiation

Mouse neuronal N1E-115 (JCRB Cell Bank of Japan Health Sciences Foundation) were cultured on Nunc’s cell culture dishes (ThermoFisher Scientific, Waltham, MA, USA) in high-glucose Dulbecco’s modified Eagle medium (DMEM; Nacalai Tesque) containing 10% heat-inactivated fetal bovine serum (ThermoFisher Scientific) and PenStrep (ThermoFisher Scientific) in 5% CO_2_ at 37 °C. To induce differentiation, N1E-115 cells were cultured in DMEM and 1% fetal bovine serum containing PenStrep in the presence or absence of 15 micromolar concentration of hesperetin (Fujifilm), excluding research on its concentration in 5% CO_2_ at 37 °C for 0 or 3 days. Cells with processes of more than three cell bodies in lengths were considered to be process-bearing cells (i.e., differentiated cells) [21,22,29]. Under these conditions, attached cells incorporating trypan blue (Nacalai Tesque) were estimated to be less than 5% in each experiment [29].

FBD-102b cells, which belong to the mouse oligodendroglial precursor cell line, were cultured on cell culture dishes in DMEM/F-12 medium (Nacalai Tesque) containing 10% heat-inactivated fetal bovine serum and PenStrep in 5% CO_2_ at 37 °C. To induce differentiation, cells were cultured on polylysine (Nacalai Tesque)-coated cell culture dishes in culture medium with 1% fetal bovine serum for 0 to 3 days in 5% CO_2_ at 37 °C. Cells with secondary branches from primary ones or with myelin membrane-like widespread membranes (cells large enough to contain a circle with a diameter of ≥25 mm) were considered to represent the differentiated phenotype [29,30]. Under these conditions, attached cells incorporating trypan blue were estimated to be less than 5% in each experiment [29,30].

Cell morphologies were captured using microscopic systems equipped with i-NTER LENS (Micronet, Saitama, Japan) and i-NTER software (Ver.2.1, Micronet). The resultant images were analyzed using Image J software v1.54f (https://imagej.nih.gov/, accessed on 1 August 2023).

### 2.5. Isolation and Culture of Primary Cortical Neuronal Cells

Primary cortical neuronal cells were isolated from the cerebrum regions of mouse embryos at embryonic days 16 to 17 and cultured as previously described [31,32]. The growing cultured cortical neuronal cells underwent detachment via trypsinization once. To begin experiments for observing neuronal process (neurite) elongation, neurons were reattached to cultured dishes and allowed to elongate neurites for ≥48 h. Under these conditions, attached cells incorporating trypan blue were estimated to be less than 7.5% in each experiment.

### 2.6. siRNA Transfection

Cell lines and primary neurons were transfected with the respective synthesized 21-mer siRNAs with dTdT using the ScreenFect siRNA transfection kit (Fujifilm) in accordance with the manufacturer’s instructions. The medium was replaced 4 h after transfection and generally used for ≥48 h after transfection for biochemical experiments. Under these conditions, attached cells incorporating trypan blue were estimated to be less than 5% in each experiment 48 h after transfection.

### 2.7. Polyacrylamide Gel Electrophoresis and Immunoblotting

Cells were lysed in lysis buffer (50 mM HEPES-NaOH, pH 7.5, 150 mM NaCl, 3 mM MgCl_2_, 1 mM dithiothreitol, 1 mM phenylmethane sulfonylfluoride, 1 μg/mL leupeptin, 1 mM EDTA, 1 mM Na_3_VO_4_, 10 mM NaF, and 0.5% NP-40) [29,30]. For denatured conditions, cell lysates were denatured in sample buffers (Fujifilm). The denatured samples were separated on 10% to 15% of premade sodium dodecylsulfate-polyacrylamide gel (Nacalai Tesque or Fujifilm). The electrophoretically separated proteins were transferred to a polyvinylidene fluoride membrane (Fujifilm), blocked with Blocking One (Nacalai Tesque), and immunoblotted using primary antibodies, followed by peroxidase enzyme-conjugated secondary antibodies. The peroxidase-reactive bands were captured using CanoScan LiDE 400 (Canon, Tokyo, Japan) and scanned using CanoScan software (Ver. 1.2, Canon). We performed multiple sets of experiments in immunoblotting studies and quantified other immunoreactive bands with the control sample’s immunoreactive band as 100% with Image J software.

### 2.8. Statistical Analyses

Values are means ± standard deviation from separate experiments. Intergroup comparisons were performed using the unpaired *t*-test with the Student’s or Welch’s correction in Excel software (Microsoft, Redmond, WA, USA). For more than three samples, one-way analysis of variance (ANOVA) was followed by a Fisher’s protected least significant difference (PLSD) test as a post hoc comparison using StatPlus software (Ver. 2017, AnalystSoft, Walnut, CA, USA). Differences were considered significant at *p* < 0.05. For all analyses, the investigator was blinded to the sample conditions.

### 2.9. Ethics Statement

Techniques using genetically modified cells and related techniques were performed in accordance with a protocol approved by the Tokyo University of Pharmacy and Life Sciences Gene and Animal Care Committee (Approval Nos. LS28-20 and LSR3-011).

## 3. Results

### 3.1. Rab11a Positively Regulates Neuronal Cell Morphological Differentiation

**Figure 1:**
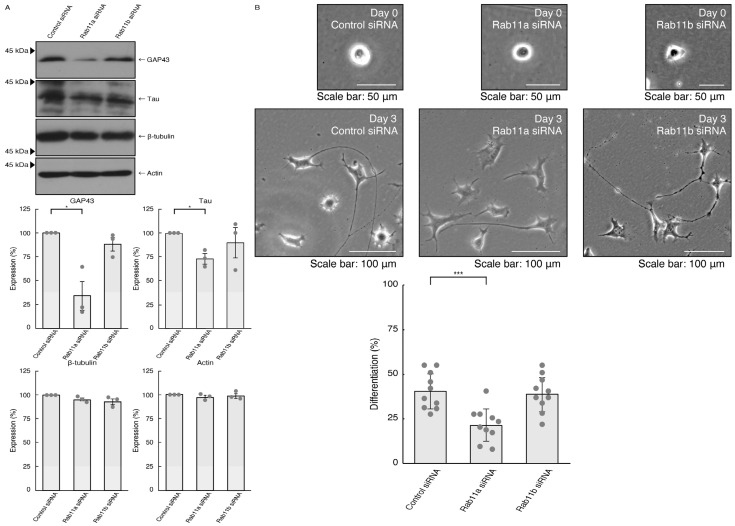
Knockdown of Rab11a and Rab11b affects neuronal cell morphological differentiation. (**A**) N1E-115 cells were transfected with control (luciferase), Rab11a, or Rab11b siRNA, allowed to differentiate for 3 days, and immunoblotted using the respective antibodies for neuronal differentiation markers (GAP43 and Tau) and internal control proteins (tubulin and actin). The respective statistical analyses with control values as 100% for scanned immunoreactive bands were depicted in the graph (* *p* < 0.05; *n* = 3 blots). (**B**) Following the induction of differentiation, the respective cell morphologies at 0 and 3 days were placed as the representative images, analyzed, and statistically depicted (*** *p* < 0.001; *n* = 10 fields). Cells with processes three times the lengths of the cell body were considered differentiated ones.

To investigate whether Rab11a, the major C9orf72 effector, is involved in the regulation of neuronal cell morphological differentiation, we knocked down Rab11a in N1E-115 cells as the differentiation model cells. The knockdown of Rab11a (see Figure S1), but not Rab11b (see Figure S2), Rab11a homolog, resulted in a decrease in the expression levels of neuronal differentiation marker proteins growth-/growth cone-associated protein 43 (GAP43) and Tau (Figure 1A). In contrast, tubulin and actin proteins, as the internal controls, were comparable in cells transfected with control siRNA or Rab11a siRNA. Consistently, the elongation of neurite-like processes as differentiated morphologies was decreased following the knockdown of Rab11a but not Rab11b (Figure 1B), suggesting that Rab11a specifically regulates neuronal cell type morphological differentiation.

We investigated whether Rab11a is involved in the regulation of neurite elongation in primary neurons. We transfected Rab11a or control siRNA into primary cortical neurons and the cells were cultured to observe neurite elongation. The knockdown of Rab11a led to decreased neurite elongation, while control knockdown resulted in the elongation of neurites (Panel A of Figure S3). These findings suggest that Rab11a promotes neuronal morphological differentiation.

**Figure 2:**
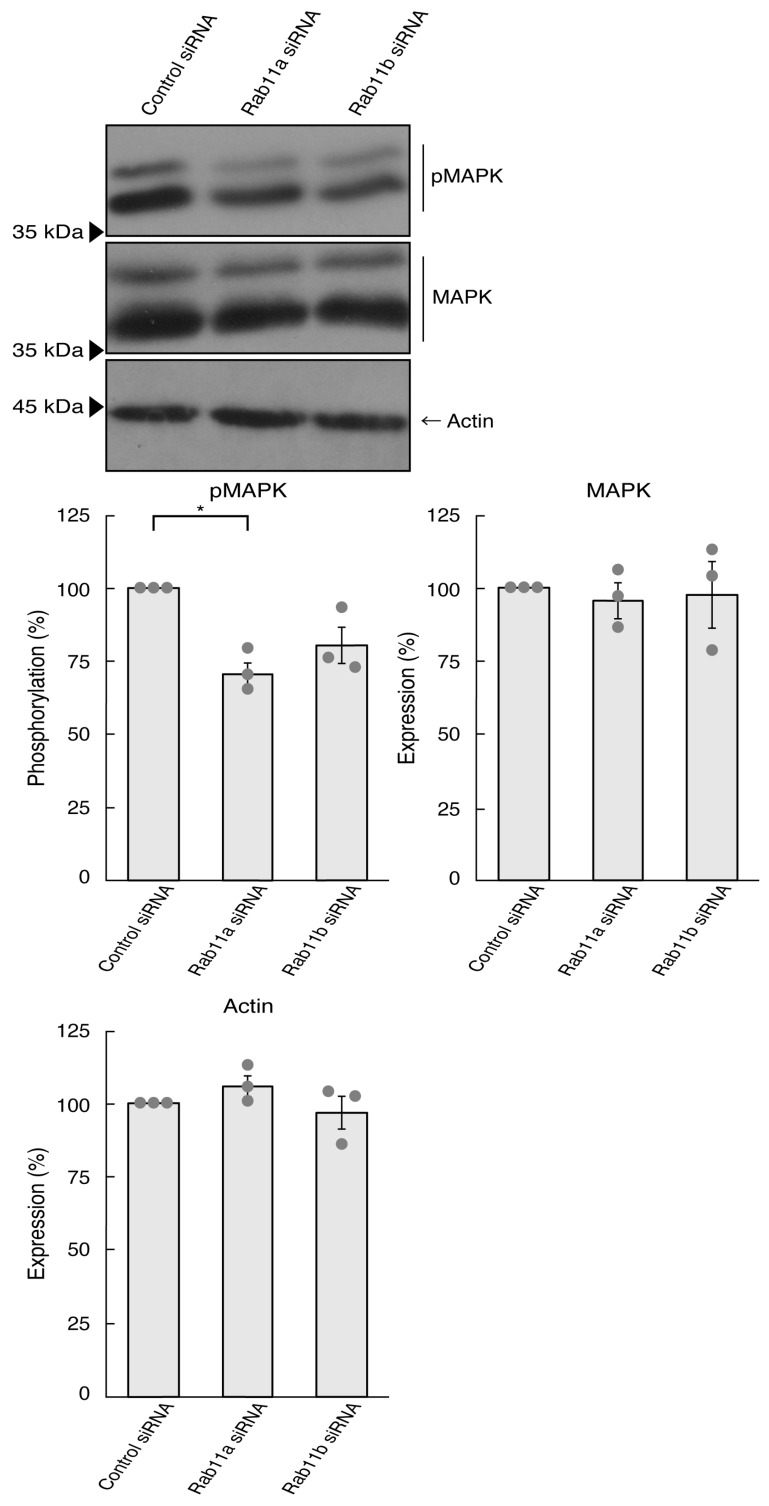
Knockdown of Rab11a and Rab11b decreases MAPK phosphorylation. N1E-115 cells were transfected with control (luciferase) Rab11a, or Rab11b siRNA, allowed to differentiate for 3 days, and immunoblotted using the respective antibodies (phosphorylated 42 kDa and 44 kDa MAPK proteins [pMAPK], total 42 kDa and 44 kDa MAPK proteins [MAPK], and actin). The respective statistical analyses were depicted in the graph (* *p* < 0.05; *n* = 3 blots).

It is well known that signaling through MAPK/ERK is critical to the mediation of many biological functions, including neuronal process elongation [27,28,29]. It is believed that MAPK phosphorylation is recognized as one of the neuronal morphological differentiation markers. We thus asked whether the knockdown of Rab11a affects MAPK phosphorylation. Since phosphorylation levels are equally correlated with the activities [27,28,29], we performed immunoblotting with an anti-phosphorylated MAPK antibody. The knockdown of Rab11a decreased the phosphorylation levels of MAPK (Figure 2). In contrast, the levels of MAPK proteins themselves were comparable. In addition, Rab11b knockdown tended to decrease the phosphorylation levels. This might result from Rab11b being involved in the signaling pathway activating MAPK. Alternatively, it might be due to multiple functions of MAPK in cells. In either case, it is clear that Rab11a can, at least in part, promote process elongation through MAPK.

### 3.2. Hesperetin Recovers Cellular Phenotypes Induced by Rab11a Knockdown

Hesperetin, a citrus flavonoid, has demonstrated neuroprotective effects in neurological diseases such as Parkinsonism, including Parkinson’s disease [23,24,25,26]. Its impact extends to dementia, including Alzheimer’s disease [23,24,25,26], in indicating neuroprotective properties. Given these observations, it prompts consideration that hesperetin could potentially restore cellular phenotypes knocked down by siRNA for Rab11a as the major C9orf72 effector. In addition, hesperetin has a wide range of protective effects on CNS diseases beyond Alzheimer’s and Parkinson’s diseases [23,24,25,26]. Therefore, to clarify whether hesperetin has a positive effect on neuronal morphological differentiation, we examined its effects in a time- and concentration-dependent manner. The results revealed that the effect of hesperetin was indeed time- and concentration-dependent (Figure S4).

**Figure 3:**
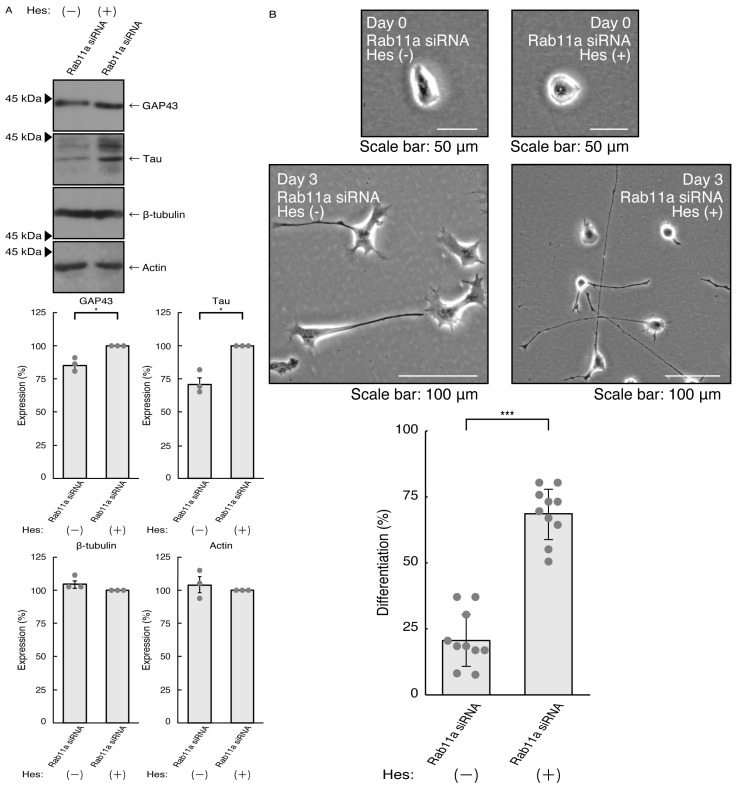
Hesperetin recovers knocking down the effects of Rab11a. (**A**) N1E-115 cells were transfected with Rab11a siRNA, treated with 15 micromolar concentration of hesperetin (Hes [+]) or its vehicle control (Hes [−]), allowed to differentiate for 3 days, and immunoblotted with the respective antibodies for neuronal differentiation markers (GAP43 and Tau) and internal control proteins (tubulin and actin). The respective statistical analyses are depicted in the graph (* *p* < 0.05; *n* = 3 blots). (**B**) Following the induction of differentiation, the respective cell morphologies at 0 and 3 days are placed as the representative images, analyzed, and statistically depicted (*** *p* < 0.001; *n* = 10 fields).

**Figure 4:**
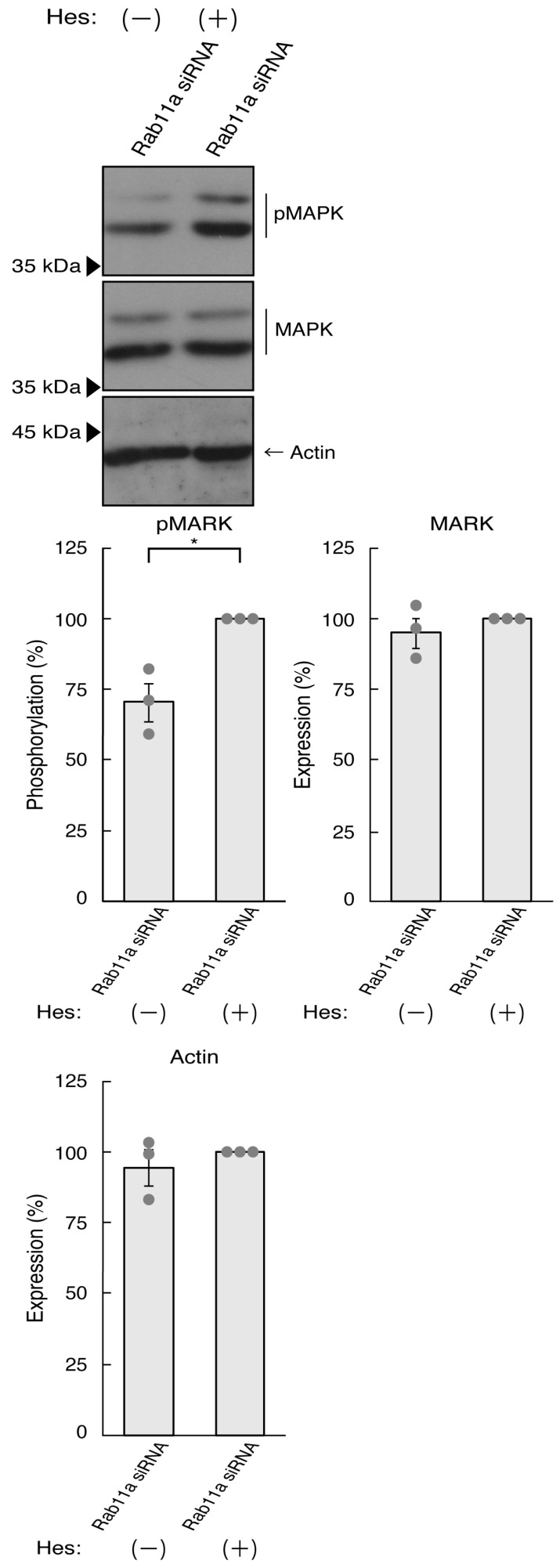
Hesperetin recovers MAPK phosphorylation decreased by knocking down Rab11a. N1E-115 cells were transfected with Rab11a siRNA, treated with 15 micromolar concentration of hesperetin (Hes [+]) or its vehicle control (Hes [−]), allowed to differentiate for 3 days, and immunoblotted with immunoblotted with the respective antibodies (pMAPK, MAPK, and actin). The respective statistical analyses were depicted in the graph (* *p* < 0.05; *n* = 3 blots).

Thus, we treated N1E-115 cells, in which Rab11a siRNA was used for knocking down, with hesperetin and sought to assess its effects. Treatment with hesperetin recovered the expression levels of GAP43 and Tau in the cells of the Rab11a knockdown background (Figure 3A). The internal control proteins, i.e., tubulin and actin, were comparable. Consistent with these results, morphologies in Rab11a knocked down cells were recovered by treatment with hesperetin (Figure 3B). In addition, phosphorylation levels of MAPK were also recovered by treatment with hesperetin (Figure 4).

We explored whether hesperetin can reverse the effects of knocking down Rab11a in primary neurons. Hesperetin promoted neurite elongation (Panel B of Figure S3), suggesting that hesperetin has similar effects on primary cortical neurons as observed in N1E-115 cells.

### 3.3. Rab11a and Rab11b Also Positively Regulate Oligodendroglial Cell Morphological Differentiation

**Figure 5:**
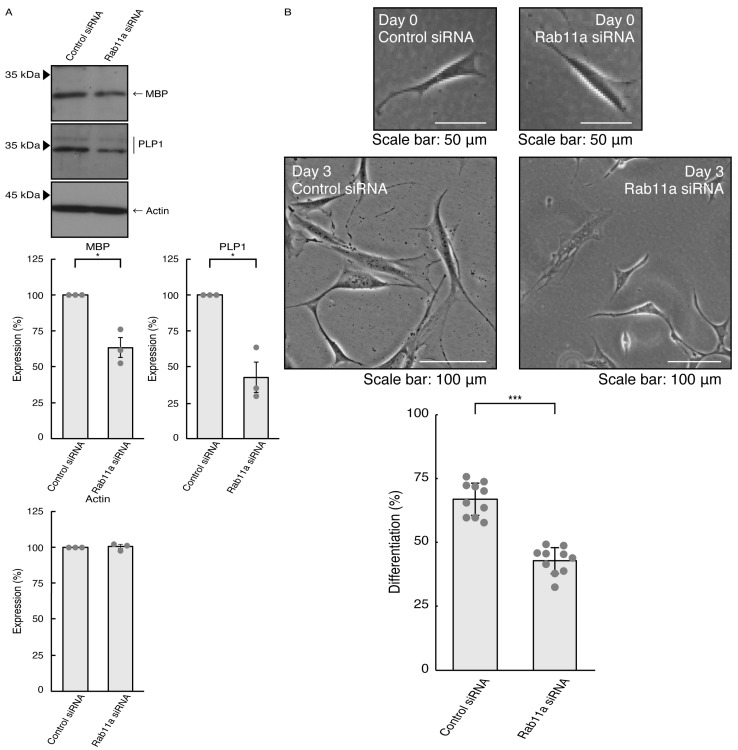
Knockdown of Rab11a affects oligodendroglial cell morphological differentiation. (**A**) FBD-102b cells were transfected with control (luciferase) or Rab11a siRNA, allowed to differentiate for 3 days, and immunoblotted with the respective antibodies for oligodendroglial differentiation markers (MBP and PLP1) and internal control protein (actin). The respective statistical analyses were depicted in the graph (* *p* < 0.05; *n* = 3 blots). (**B**) Following the induction of differentiation, the respective cell morphologies at 0 and 3 days were placed as the representative images, analyzed, and statistically depicted (*** *p* < 0.001; *n* = 10 fields). Cells with secondary branches or cell bodies large enough to accommodate a 25-micrometer circle were considered differentiated ones.

**Figure 6:**
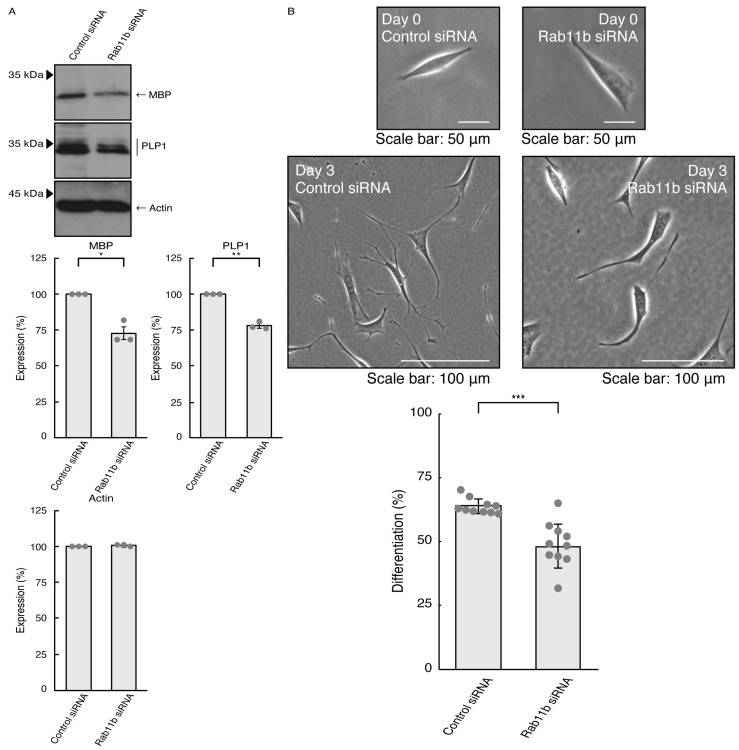
Knockdown of Rab11b affects oligodendroglial cell morphological differentiation. (**A**) FBD-102b cells were transfected with control (luciferase) or Rab11b siRNA, allowed to differentiate for 3 days, and immunoblotted with the respective antibodies for oligodendroglial differentiation markers (MBP and PLP1) and internal control protein (actin). The respective statistical analyses were depicted in the graph (** *p* < 0.01 and * *p* < 0.05; *n* = 3 blots). (**B**) Following the induction of differentiation, the respective cell morphologies at 0 and 3 days were placed as the representative images, analyzed, and statistically depicted (*** *p* < 0.001; *n* = 10 fields).

Given that Rab11a as the C9orf72 effector may be involved in the formation of neuronal cell type morphologies, we predicted that Rab11a might also participate in differentiation in oligodendroglial cells. We knocked down Rab11a (see Figure S5) in the FBD-102b cells as the differentiation model of oligodendroglial cells. The knockdown of Rab11a (see Figure S6) decreased the expression levels of oligodendroglial differentiation marker proteins, i.e., myelin basic protein (MBP) and proteolipid protein (PLP1), but not internal control actin protein (Figure 5A). Rab11a knockdown also decreased protrusion complexities in cells (Figure 5B), indicating that Rab11a is positively involved in the regulation of oligodendroglial cell morphological differentiation. Similar results in marker protein expression levels and protrusion complexities were obtained in the case of Rab11b knockdown (Figure 6A,B). Despite the unidentified role of Rab11b downstream of C9orf72, both Rab11a and Rab11b positively regulate oligodendroglial cell morphological differentiation. Taken together, our results suggest that Rab11a, which acts downstream of C9orf72, is more specialized in neuronal cell morphological differentiation. Furthermore, its knockdown effect is restored by hesperetin.

### 3.4. Knockdown of Rab14 Does Not Significantly Affect Cell Morphogenesis

Lastly, we examined whether Rab14 [17,18,19,20], a molecule homologous to Rab11a and Rab11b, is involved in the regulation of cell morphological differentiation. The knockdown of Rab14 (Figures S7 and S8) had no effects on the expression levels of neuronal differentiation markers or the cell morphologies in N1E-115 cells (Figure S9). Additionally, in FBD-102b cells, Rab14 knockdown (see Figure S8) affected neither the expression levels of oligodendroglial differentiation markers nor the cell morphologies (Figure S10), suggesting the specific involvement of Rab11a of Rab11 subfamily molecules in neuronal cell morphological differentiation, together with the data of Figures S9–S18).

## 4. Discussion

FTD and/or ALS are now considered to be a single-spectrum disorder, although FTDALS is a heterogeneous disease with different clinical phenotypes associated with multiple neuropathologic effects [7,8,9,10,11,12]. It is thought that FTDALS affects 1 in 50,000 people [7,8,9,10,11,12]. Thus, FTDALS is currently classified according to the responsible genes whose products are C9orf72, coiled-coil-helix-coiled-coil-helix domain containing 10 (CHCHD10), sequestosome 1 (SQSTM1), Tank-binding kinase 1 (TBK1), Cyclin F, valosin containing protein (VCP), charged multivesicular body protein 2B (CHMP2B), and lysine 63 deubiquitinase (CYLD) for FTDALS1, FTDALS2, FTDALS3, FTDALS4, FTDALS5, FTDALS6, FTDALS7, and FTDALS8, respectively [7,8,9,10,11,12]. Through the identification and subsequent characterization of these responsible genes, it is predicted that one major molecular pathological mechanism of FTDALS links to abnormality or deficiency of proteostasis [13,14,15,16].

Since proteostasis is thought to be composed of an essential metabolic, and sometimes catabolic, pathway, its regulation is critically associated with cell determination, cell homeostasis, and morphological differentiation [15,16]. Thus, we investigated whether the activities of the C9orf72 protein, specifically the regulatory effects of Rab proteins serving as downstream effectors in mediating proteostasis, actually regulate morphological changes in neuronal cells. We found that the knockdown of Rab11a, one of the major C9orf72 effectors, significantly leads to decreasing the elongation of neurite-like processes. In contrast, the knockdown of Rab11b does not significantly affect their elongation, suggesting that the C9orf72 protein unlikely affects such morphological differentiation. Although the link between Rab11a deficiency and neuronal degradation remains to be clarified, it is clear that Rab11a, a Rab11 subfamily molecule, is required to achieve morphological differentiation. It is possible that Rab11a is also needed to maintain cell morphologies. If this is true, Rab11a might be one of the reasons why the loss of function of C9orf72 causes the neurodegradative phenotypes observed in FTDALS1 at the molecular and cellular levels. Additionally, Rab11a may participate not only in neuronal process elongation and formation but also in the formation of branches from its major processes. Also, Rab11a may be at least partially involved in certain regenerating steps of neuronal processes following breakdown due to physical and physiological stresses.

It remains to be elucidated which molecule in the cells is the target of hesperetin that confers protective effects on phenotypes induced by Rab11a knockdown. The target might be an upstream molecule of Rab11a. There are some GEFs for Rab11a as the upstream Rab11a activator. First, FLCN (also called folliculin or DENND8B), the gene product responsible for Birt–Hogg–Dubé syndrome, is characterized to be the GEF specific for Rab11a [33,34]. FLCN is known to be located within the Smith–Magenis syndrome region on chromosome 17 [33,34]. Individuals with Smith–Magenis syndrome often exhibit distinctive facial appearance, delayed mental development, congenital heart disease, intractable epilepsy, and behavioral abnormalities. It is thus predicted that FLCN plays a role in cell and tissue morphogenesis in some organs, as well as the CNS, suggesting that the FLCN and Rab11a signaling cascade is involved in the regulation of cell morphogenesis. In addition, FLCN directly interacts with 43kDa DNA binding protein (TDP-43) whose mutations are associated with ALS with or without FTD [35,36,37,38,39,40]. Second, Src homology domain 3 binding protein (SH3BP5) acts as the GEFs for Rab11 and Rab11b as well as Rab14 [39,40]. SH3BP5 lacks the DENN domain. Although it is unclear whether SH3BP5 positively controls cell morphogenesis, it has neuroprotective effects through certain secreted bioactive peptides [41,42,43]. Third, it is likely that guanine nucleotide exchange factor for Rab3A (GRAB, also called Rab3il1) directly interacts with Rab11a and Rab11b and acts to promote the exchange reaction for Rab11a and Rab11b [44]; however, it does not display moderate to high expression levels in neuronal tissues (see the Gene website, https://www.ncbi.nlm.nih.gov/gene/74760, accessed on 1 August 2023). Together with the genetic and biochemical studies described above, it is conceivable that FLCN and SH3BP5, as well as C9orf72, constitute the signaling pathway coupling to Rab11a to regulate neuronal cell morphological differentiation properly. If its activation of FLCN and/or SH3BP5 by chemical compounds such as hesperetin could be controlled when C9orf72 activity is stalled, it might be possible to promote morphological differentiation and/or to maintain differentiating states.

The targets of hesperetin may include a molecule acting downstream of Rab11a. More than 800 types of molecules have been identified as binding partners with Rab11a biochemically and/or by using proteome analyses (see the BioGrid website, https://thebiogrid.org). Among them, it is of note that Rab11a and Rab11b bind to molecules composed of the exocyst complex controlling exocytosis, although the exocyst complex is not sufficiently specific for Rab11 proteins [45,46]. One of the key reasons may be due to transport molecules controlling morphological changes to the plasma membrane or sometimes the lysosome involved in proteostasis [45,46]. For example, Exoc2, a subunit of the exocyst complex, is associated with neurodevelopmental disorder with dysmorphic facies and cerebellar hypoplasia (NEDFACH). The loss of function of Exoc2 via the nonsense mutation in Arg at the 437 position causes severe defects in the development of the human brain, indicating that the loss of function of Exoc2 is critically linked to defective cell morphological change [45,46]. Exoc7 and Exoc8, subunits of the exocyst complex, are also associated with neurodevelopmental disorders with microcephaly, seizures, and brain atrophy (NEDMISB) [45,46]. Despite the unidentified specific function of Rab11a and Rab11b in the exocyst complex, Rab11a as the effector of C9orf72 may act upstream of exocyst proteins Exoc2, Exoc7, and Exoc8 and regulates neuronal cell morphological differentiation. Additionally, since it is well known that focal adhesion kinase (FAK) and Akt act as the further downstream molecules of Rab11a [47,48,49,50], future research will allow us to clarify the relationships of other effector molecules including Exoc2, Exoc7, and Exoc8 with hesperetin.

It is likely that FTDALS diseases including FTDALS1 affect not only neuronal cells but also oligodendroglial cells [51,52,53,54,55]. The mutations of C9orf72 result in disrupted myelin lipid metabolism [53]. There is also a report that demyelination due to neuroinflammation is more likely to occur [54]. Here we find that Rab11a knockdown blunts oligodendroglial cell morphological differentiation. The phenotypes of the thin and/or degradative myelin sheaths observed in FTDALS1 may reflect defective oligodendroglial cell morphological differentiation. Since Rab11a has multiple functions, including intracellular vesicle trafficking [55,56,57,58], it may be reasonable that its knockdown affects oligodendroglial cell morphologies.

C9orf72 functions as the GEFs for Rab proteins other than Rab11a. Rab39b is also an important effector of C9orf72 [9,10,11,12]. Indeed, it is interesting that Rab39b can actually have an effect on cell morphologies [59,60,61,62]. However, since Rab11a has been well studied in cell types other than neuronal cells [9,10,11,12], we opted to focus our study on Rab11a as downstream of C9orf72. This is reminiscent of the relationship between Rab11a with neurite elongation and/or maintenance. Although the relationship between Rab39b with neurite elongation remains to be elucidated, the precise role of Rab39b in neuronal cells is an important issue to be addressed in future research. We herein demonstrate that the knockdown of Rab11a has an effect on neuronal cell morphological differentiation. The phenotypes are recovered via treatment with hesperetin. In addition, the knockdown of Rab11a, a C9orf72 effector, also affects oligodendroglial cell morphological differentiation. Further studies will promote our understanding of the detailed mechanism whereby the knockdown of Rab11a has inhibitory effects on morphological differentiation in neuronal and glial cells. Additional studies will also allow us to clarify how hesperetin recovers phenotypes via its knockdown at the molecular and cellular levels. These studies will help us develop therapeutic target–specific medicines for FTDALS diseases and related ones as well as the Rab11a-dependent pathological effects underlying FTDALS1 [63,64].

## Figures and Tables

**Table 1:** Key antibodies and chemicals used in this study.

Reagents or Sources	Company or Source	Cat. No.	Lot. No.	Concentration Used
Antibodies				
Ant-growth associated protein 43 (GAP43)	Santa Cruz Biotechnology (Santa Cruz, CA, USA)	sc-17790	J0920	Immunoblotting (IB), 1:20,000
Anti-tau	Santa Cruz Biotechnology	sc-21796	G1222	IB, 1:500
Anti-tubulin (beta type)	Santa Cruz Biotechnology	sc-80005	A1821	IB, 1:20,000
Anti-actin (also called beta-type actin)	MBL (Tokyo, Japan)	M177-3	007	IB, 1:5000
Anti-phospho-p44/42 MAPK (pErk1/2)	Cell Signaling Technology (Danvers, MA, USA)	4370S	5	IB, 1:2000
Anti-p44/42 MAPK (Erk1/2)	Cell Signaling Technology	4695T	35	IB, 1:1000
Anti-myelin basic protein (MBP)	BioLegend (San Diego, CA, USA)	836504	B225469	IB, 1:500
Anti-proteolipid protein 1 (PLP1)	Atlas Antibodies (Bromma, Sweden)	HPA004128	8115828	IB, 1:500
Anti-IgG (H+L chain) (mouse) pAb-HRP	MBL (Tokyo, Japan)	330	365	IB, 1:5000
Anti-IgG (H+L chain) (rabbit) pAb-HRP	MBL	458	353	IB, 1:5000
Key chemicals				
Hesperitin (Hes)	FUJIFILM Wako Pure Chemical Corporation (Tokyo, Japan)	087-10001	DLK5755	15 miclomoler (final concentration)
Dimethyl sulfoxide (DMSO)	FUJIFILM Wako Pure Chemical Corporation	047-29353	CDN0170	Less than 0.1% (final concentration)
Cell lines				
FBD-102b cells (mouse cells)	Dr. Yasuhiro Tomo-oka (Tokyo University of Science, Chiba, Japan and Riken, Saitama, Japan)	N/A	N/A	
N1E-115 cells (mouse cells)	Dr. Daisuke Shiokawa (Tokyo University of Science, Chiba, Japan)	N/A	N/A	

## Data Availability

The datasets used and/or analyzed for the current study are available from the corresponding author upon reasonable request.

## Supplementary Materials

The following supporting information can be downloaded at: https://www.mdpi.com/article/10.3390/pathophysiology31010008/s1, Figure S1. Effects of knockdown of Rab11a in N1E-115 cells; Figure S2. Effects of knockdown of Rab11b in N1E-115 cells; Figure S3. Effects of knockdown of Rab11a in primary cortical neurons; Figure S4. The relationship of hesperetin with morphological differentiation in N1E-115 cells; Figure S5. Effects of knockdown of Rab11a in FBD-102b cells; Figure S6. Effects of knockdown of Rab11b in FBD-102b cells; Figure S7. Effects of knockdown of Rab14 in N1E-115 cells; Figure S8. Effects of knockdown of Rab14 in FBD-102b cells; Figure S9. Effects of knockdown of Rab14 in N1E-115 cell morphologies; Figure S10. Effects of knockdown of Rab14 in FBD-102b cell morphologies; Figure S11. Original size gels saved as Figure 1 TIFF files; Figure S12. Original size gels saved as Figure 2 TIFF files; Figure S13. Original size gels saved as Figure 3 TIFF files; Figure S14. Original size gels saved as Figure 4 TIFF files; Figure S15. Original size gels saved as Figure 5 TIFF files; Figure S16. Original size gels saved as Figure 6 TIFF files; Figure S17. Original size gels saved as Figure S9 TIFF files; Figure S18. Original size gels saved as Figure S10 TIFF files.
