# Supplementary material for: Rab11a Controls Cell Shape via C9orf72 Protein: Possible Relationships to Frontotemporal Dementia/Amyotrophic Lateral Sclerosis (FTDALS) Type 1
Source: Pathophysiology. 2024 Feb 9;31(1):100–16. doi: 10.3390/pathophysiology31010008 (PMC10885063; doi:10.3390/pathophysiology31010008)
Supplement: Supplementary file 1 [file pathophysiology-31-00008-s001.zip › pathophysiology-2789015-supplementary.pdf]

A

siRNA sequences
siRab11a 119: 5' GCAAGAGUACCAUUGGAGU-dTdT 3'
siRab11a 123: 5' GAGUACCAUUGGAGUAGAG-dTdT 3'
siRab11a 152: 5' GAAGCAUCCAGGUUGAUGG-dTdT 3'
**PCR primers**
Rab11a (sense, Tm values = 62°C) 5' ATGGGCACCCGCGACG 3'
Rab11a (antisense, Tm values = 62°C) 5' TTAGATGTTCTGACAGCACTGCACCTTTG 3'
Actin (sense, Tm values = 66°C) 5' ATGGATGACGATATCGCTGCGCTGGTC 3'
Actin (antisense, Tm values = 66°C) 5' CTAGAAGCACTTGCGGTGCACGATGGAG 3'

B

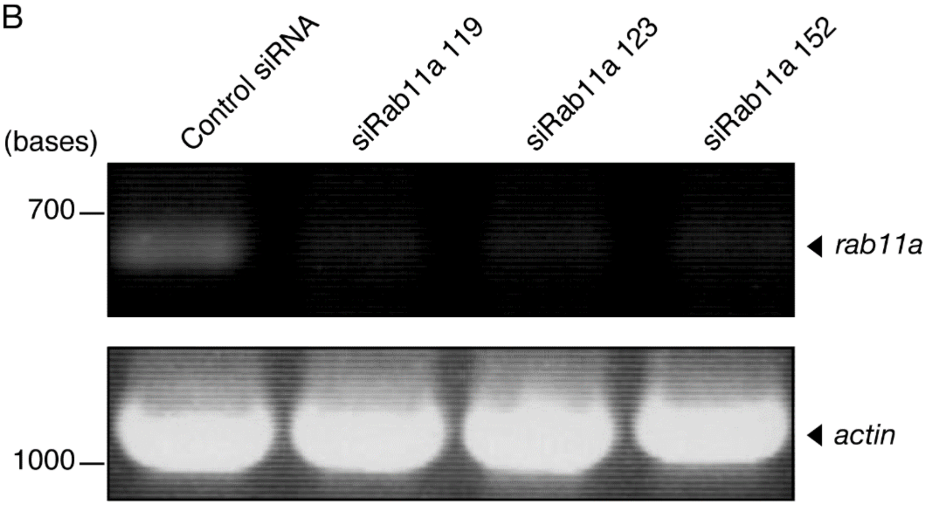

Figure S1. Effects of knockdown of Rab11a in N1E-115 cells. (A, B) Sequences of siRNAs and PCR primers. Knockdown efficiencies in transfected cells (mouse N1E-115 cell line) were determined by RT-PCR and agarose gel electrophoresis. The siRNA with sequence starting at position 119 in Rab11a was used in subsequent experiments.

A

siRNA sequences
siRab11b 123: 5’ GAGUACCAUCGGAGUGGAG-dTdT 3’
siRab11b 172: 5’ GACCAUCAAGGCUCAGAUC-dTdT 3’
siRab11b 183: 5’ GGCUCAGAUCUGGGACACU-dTdT 3’
**PCR primers**
Rab11b (sense, Tm values = 62°C) 5’ ATGGGGACCCGGGACGAC 3’
Rab11b (antisense, Tm values = 62°C) 5’ TCACAGGCTCTGGCAGCACTG 3’
Actin (sense, Tm values = 66°C) 5’ ATGGATGACGATATCGCTGCGCTGGTC 3’
Actin (antisense, Tm values = 66°C) 5’ CTAGAAGCACTTGCGGTGCACGATGGAG 3’

B

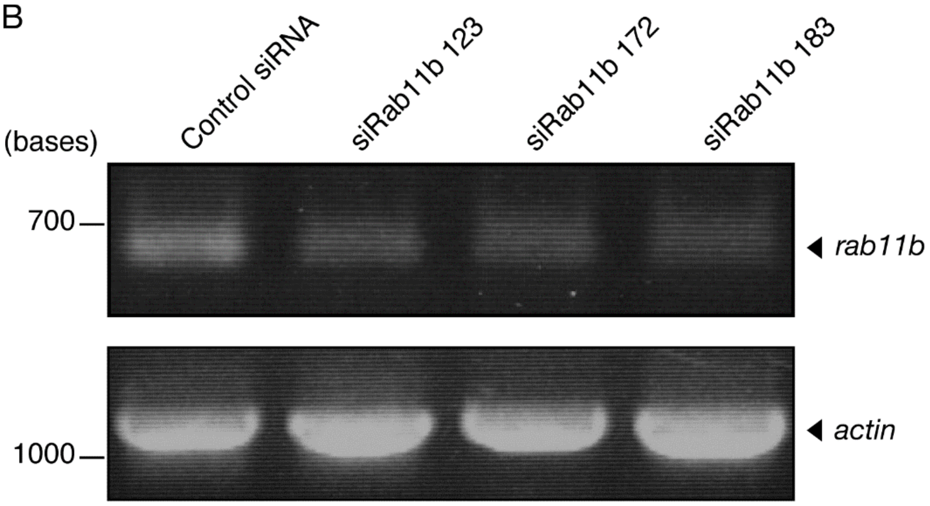

Figure S2. Effects of knockdown of Rab11b in N1E-115 cells. (A, B) Sequences of siRNAs and PCR primers. Knockdown efficiencies in transfected cells were determined by RT-PCR and agarose gel electrophoresis. The siRNA with sequence starting at position 123 in Rab11b was used in subsequent experiments.

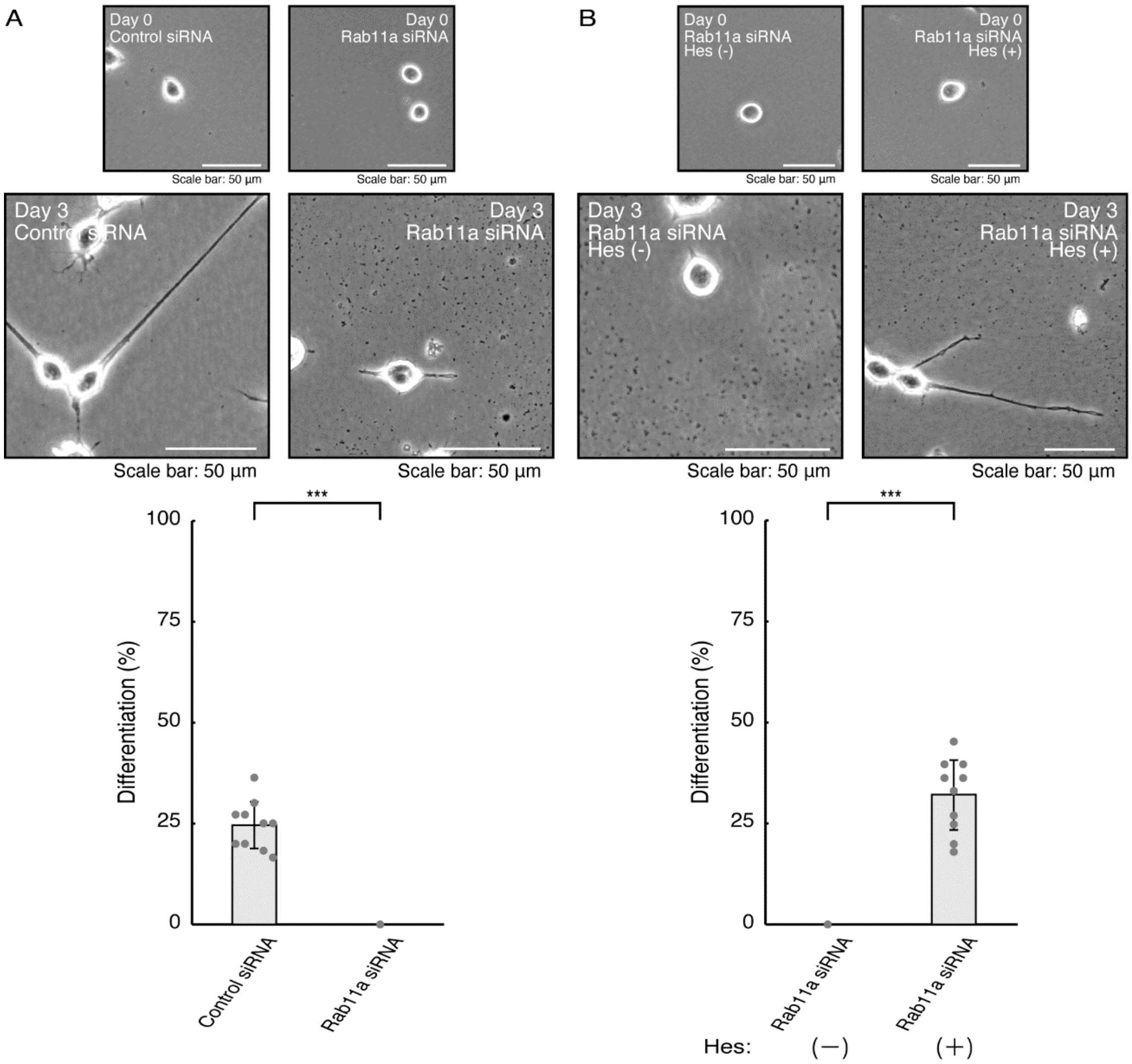

Figure S3. Effects of knockdown of Rab11a in primary cortical neurons. (A) Primary cortical neurons, isolated from embryonic brains, were transfected with control or Rab11a siRNA and allowed to elongate neurites for several days. Cells with processes three times the lengths of the cell body were considered differentiated and statistically depicted in the graph (** p<0.01; n = 10 fields). (B) Neurons were transfected with Rab11a siRNA and cultured in the presence or absence of 15 micromolar concentration of hesperetin for several days. Cells with processes were statistically depicted in the graph (** p<0.01; n = 10 fields).

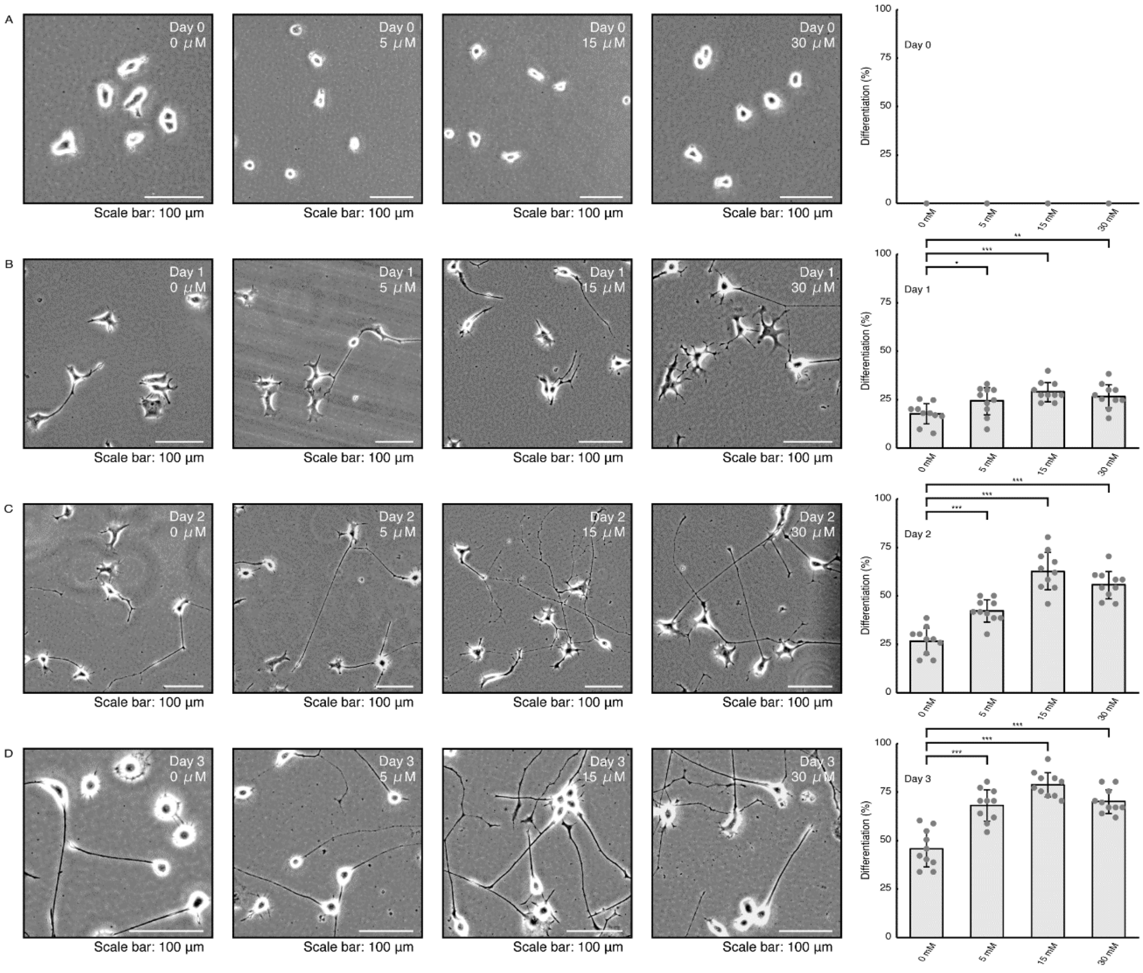

Figure S4. The relationship of hesperetin with morphological differentiation in N1E-115 cells. Cells were allowed to differentiate for 0 (A), 1 (B), 2 (C), or 3 (D) days in the presence or absence of 0 to 30 micromolar concentration of hesperetin. Cells with processes three times the lengths of the cell body were considered differentiated and statistically depicted in the graph (*** p<0.001, ** p<0.01, * p<0.05; n = 10 fields).

A

siRNA sequences
siRab11a 119: 5' GCAAGAGUACCAUUGGAGU-dTdT 3'
siRab11a 123: 5' GAGUACCAUUGGAGUAGAG-dTdT 3'
siRab11a 152: 5' GAAGCAUCCAGGUUGAUGG-dTdT 3'
**PCR primers**
Rab11a (sense, Tm values = 62°C) 5' ATGGGCACCCGCGACG 3'
Rab11a (antisense, Tm values = 62°C) 5' TTAGATGTTCTGACAGCACTGCACCTTTG 3'
Actin (sense, Tm values = 66°C) 5' ATGGATGACGATATCGCTGCGCTGGTC 3'
Actin (antisense, Tm values = 66°C) 5' CTAGAAGCACTTGCGGTGCACGATGGAG 3'

B

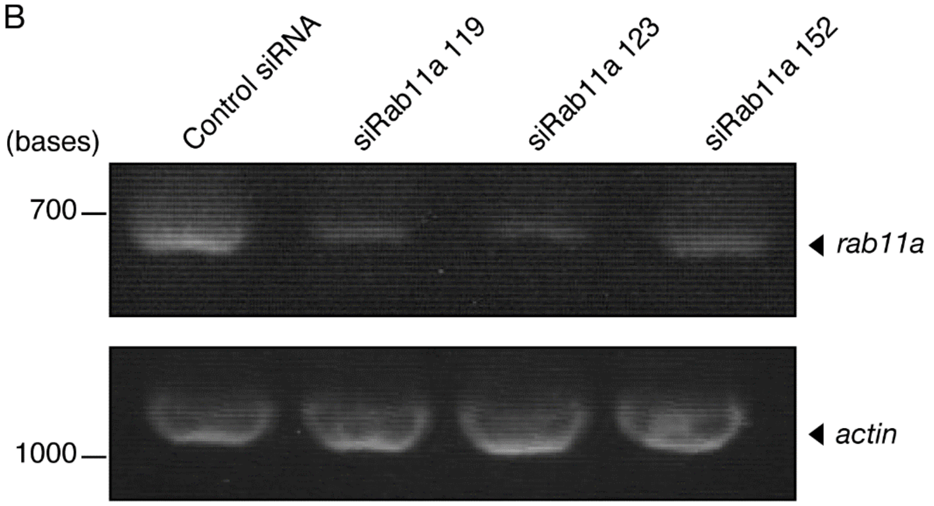

Figure S5. Effects of knockdown of Rab11a in FBD-102b cells. (A, B) Sequences of siRNAs and PCR primers. Knockdown efficiencies in transfected cells (mouse FBD-102b cell line) were determined by RT-PCR and agarose gel electrophoresis. The siRNA with sequence starting at position 119 in Rab11a was used in subsequent experiments.

A

siRNA sequences
siRab11b 123: 5’ GAGUACCAUCGGAGUGGAG-dTdT 3’
siRab11b 172: 5’ GACCAUCAAGGCUCAGAUC-dTdT 3’
siRab11b 183: 5’ GGCUCAGAUCUGGGACACU-dTdT 3’
**PCR primers**
Rab11b (sense, Tm values = 62°C) 5’ ATGGGGACCCGGGACGAC 3’
Rab11b (antisense, Tm values = 62°C) 5’ TCACAGGCTCTGGCAGCACTG 3’
Actin (sense, Tm values = 66°C) 5’ ATGGATGACGATATCGCTGCGCTGGTC 3’
Actin (antisense, Tm values = 66°C) 5’ CTAGAAGCACTTGCGGTGCACGATGGAG 3’

B

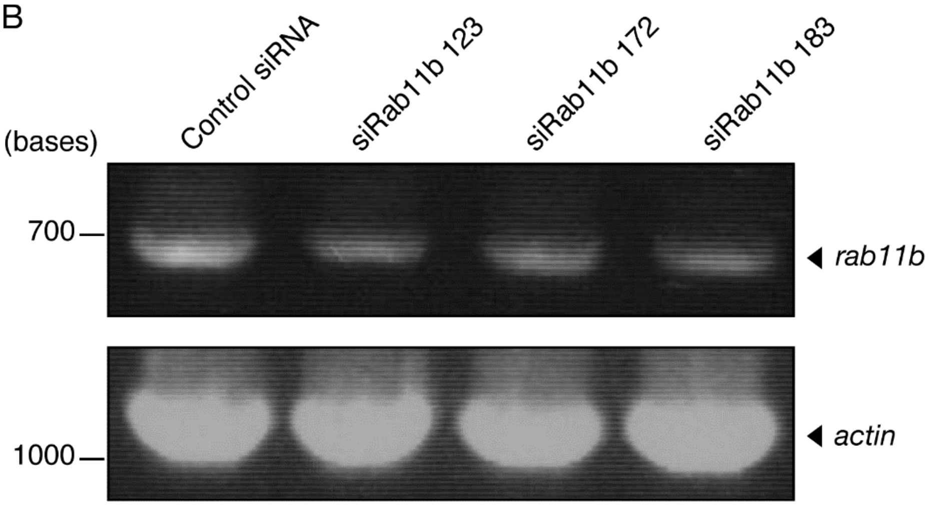

Figure S6. Effects of knockdown of Rab11b in FBD-102b cells. (A, B) Sequences of siRNAs and PCR primers. Knockdown efficiencies in transfected cells were determined by RT-PCR and agarose gel electrophoresis. The siRNA with sequence starting at position 123 in Rab11b was used in subsequent experiments.

A

siRNA sequences
siRab14 152: 5’ GAAUAAUUGAAGUUAGUGG-dTdT 3’
siRab14 163: 5’ GUUAGUGGUCAAAAAAUCA-dTdT 3’
siRab14 287: 5’ GAAGUACAUAUAACCACUU-dTdT 3’
**PCR primers**
Rab14 (sense, Tm values = 62°C) 5’ ATGGCAACTGCACCGTACAACTACTC 3’
Rab14 (antisense, Tm values = 62°C) 5’ CTAGCAGCCACAGCCTTCTCTCTG 3’
Actin (sense, Tm values = 66°C) 5’ ATGGATGACGATATCGCTGCGCTGGTC 3’
Actin (antisense, Tm values = 66°C) 5’ CTAGAAGCACTTGCGGTGCACGATGGAG 3’

B

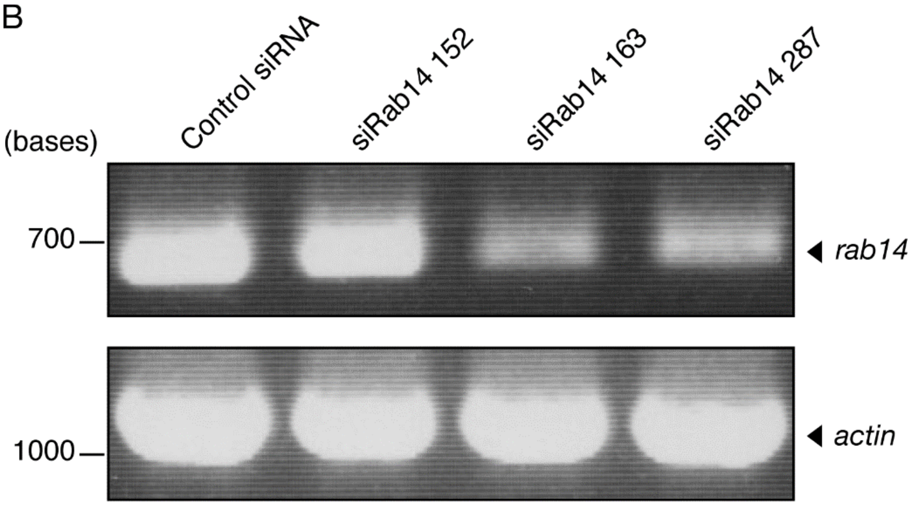

Figure S7. Effects of knockdown of Rab14 in N1E-115 cells. (A, B) Sequences of siRNAs and PCR primers. Knockdown efficiencies in transfected cells were determined by RT-PCR and agarose gel electrophoresis. The siRNA with sequence starting at position 287 in Rab14 was used in subsequent experiments.

A

siRNA sequences
siRab14 152: 5’ GAAUAAUUGAAGUUAGUGG-dTdT 3’
siRab14 163: 5’ GUUAGUGGUCAAAAAAUCA-dTdT 3’
siRab14 287: 5’ GAAGUACAUAUAACCACUU-dTdT 3’
**PCR primers**
Rab14 (sense, Tm values = 62°C) 5’ ATGGCAACTGCACCGTACAACTACTC 3’
Rab14 (antisense, Tm values = 62°C) 5’ CTAGCAGCCACAGCCTTCTCTCTG 3’
Actin (sense, Tm values = 66°C) 5’ ATGGATGACGATATCGCTGCGCTGGTC 3’
Actin (antisense, Tm values = 66°C) 5’ CTAGAAGCACTTGCGGTGCACGATGGAG 3’

B

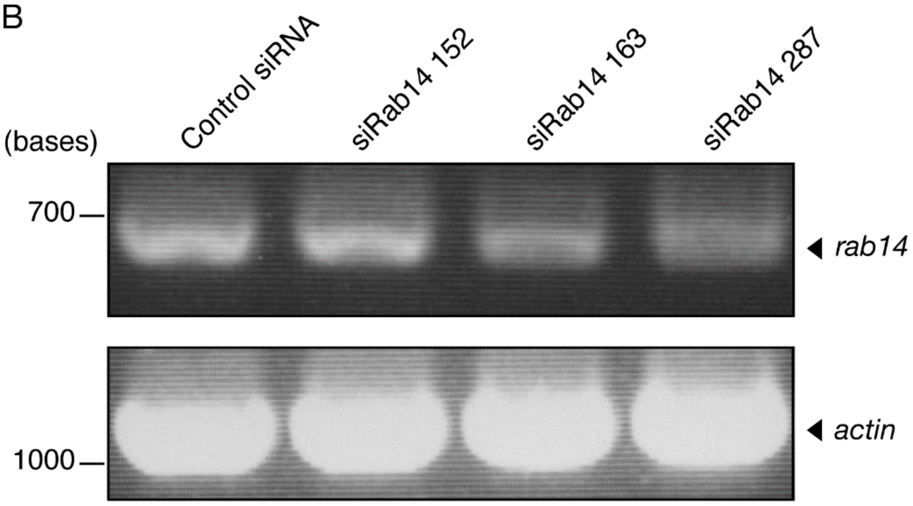

Figure S8. Effects of knockdown of Rab14 in FBD-102b cells. (A, B) Sequences of siRNAs and PCR primers. Knockdown efficiencies in transfected cells were determined by RT-PCR and agarose gel electrophoresis. The siRNA with sequence starting at position 287 in Rab14 was used in subsequent experiments.

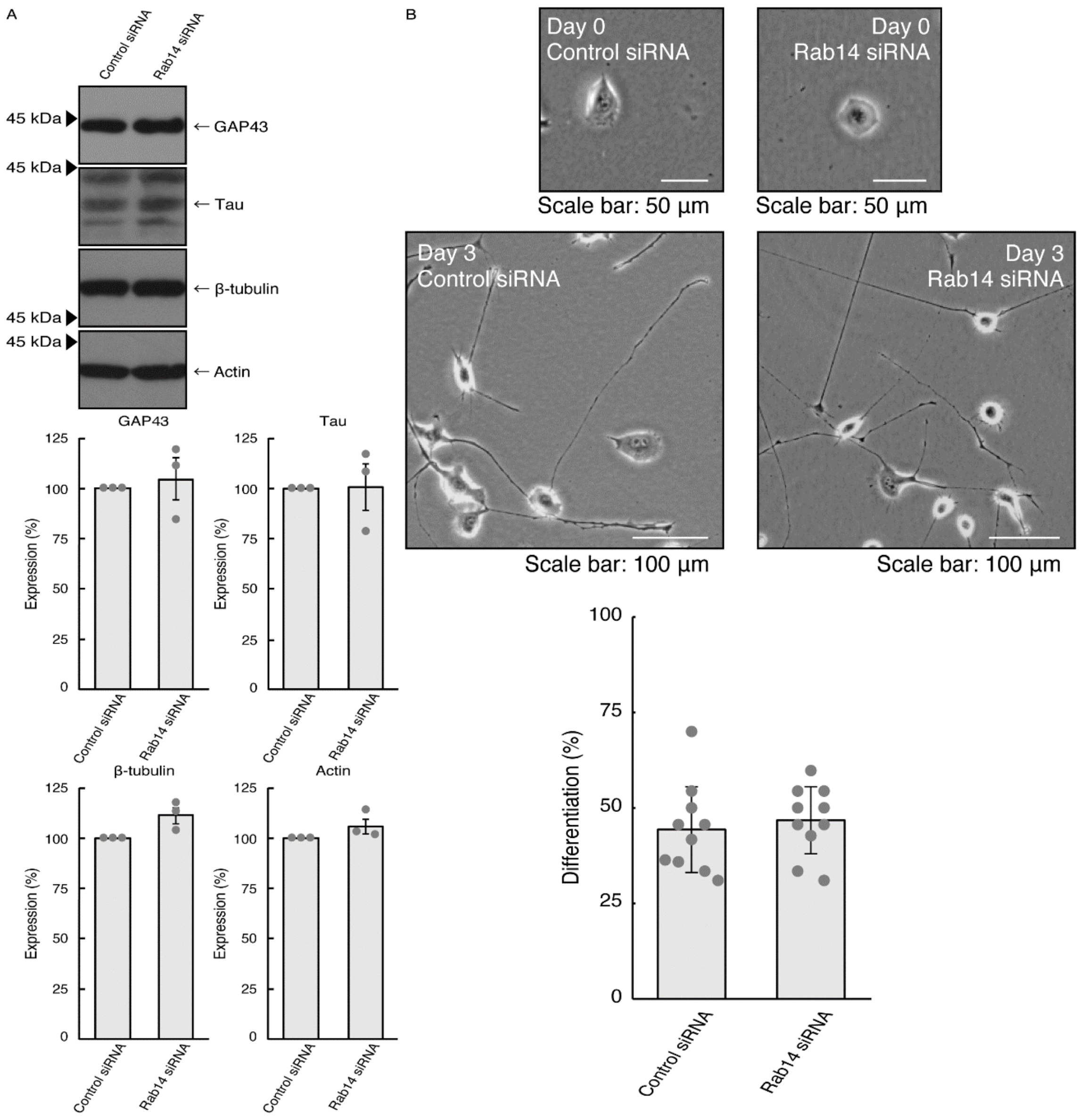

Figure S9. Effects of knockdown of Rab14 in N1E-115 cell morphologies. (A) Cells were transfected with Rab14 siRNA and immunoblotted with the respective antibodies for neuronal differentiation marker and internal control proteins. The respective statistical analyses with control values as 100% for scanned immunoreactive bands were depicted in the graph (n = 3 blots). (B) Cells were allowed to differentiate for 0 or 3 days. The respective representative images of cell morphologies were placed as grayscale ones.

Cells with processes three times the lengths of the cell body were considered differentiated ones and statistically depicted in the graph (n = 10 fields).

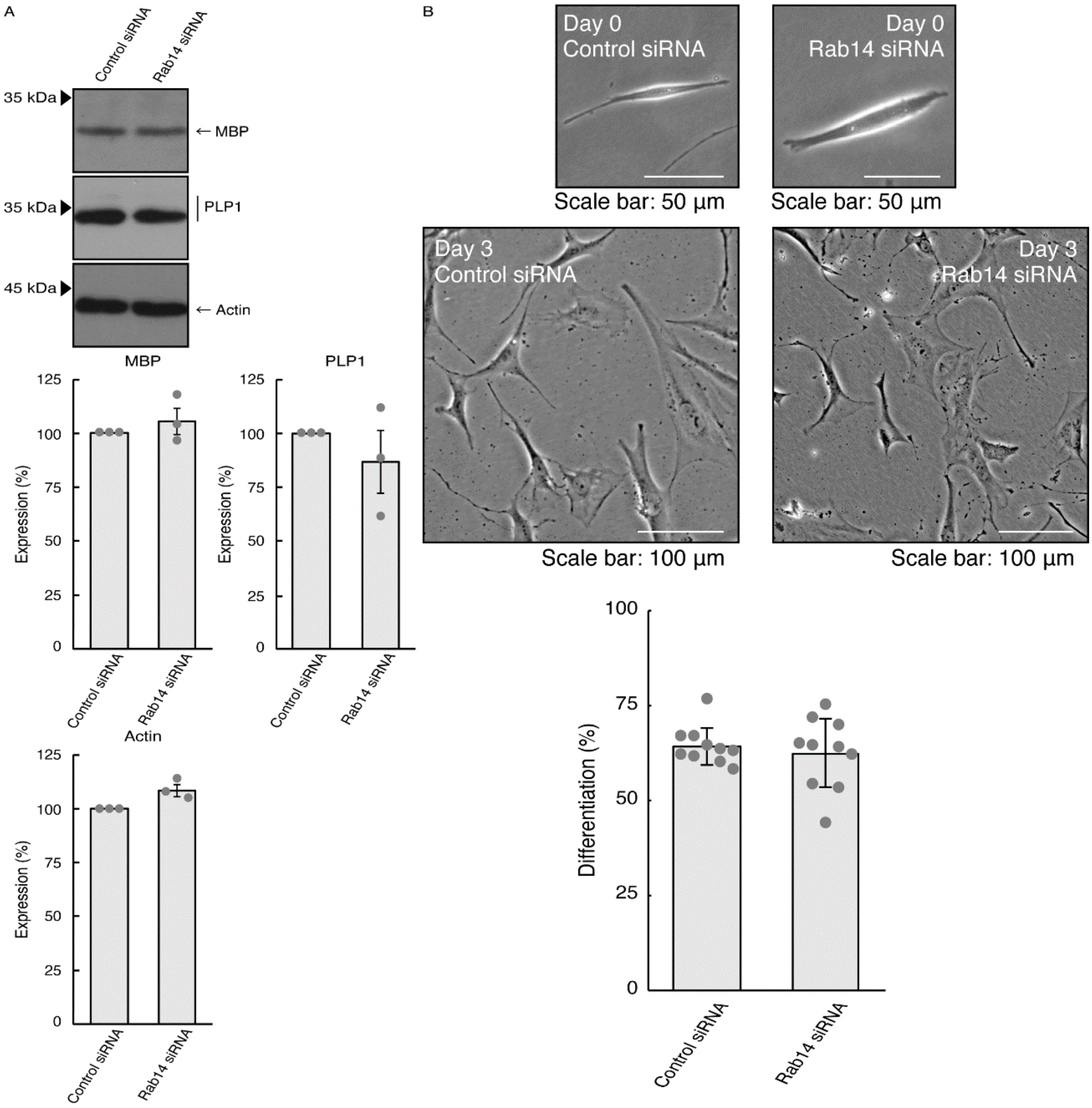

Figure S10. Effects of knockdown of Rab14 in FBD-102b cell morphologies. (A) Cells were transfected with Rab14 siRNA and immunoblotted with the respective antibodies for oligodendroglial differentiation marker and internal control proteins. The respective statistical analyses with control values as 100% were depicted in the graph (n = 3 blots). (B) Cells were allowed to differentiate for 0 or 3 days. The respective representative

images of cell morphologies were placed as grayscale ones. Cells with secondary branches or cell body large enough to accommodate a 25 micrometer circle were considered differentiated ones and statistically depicted in the graph (n = 10 fields).

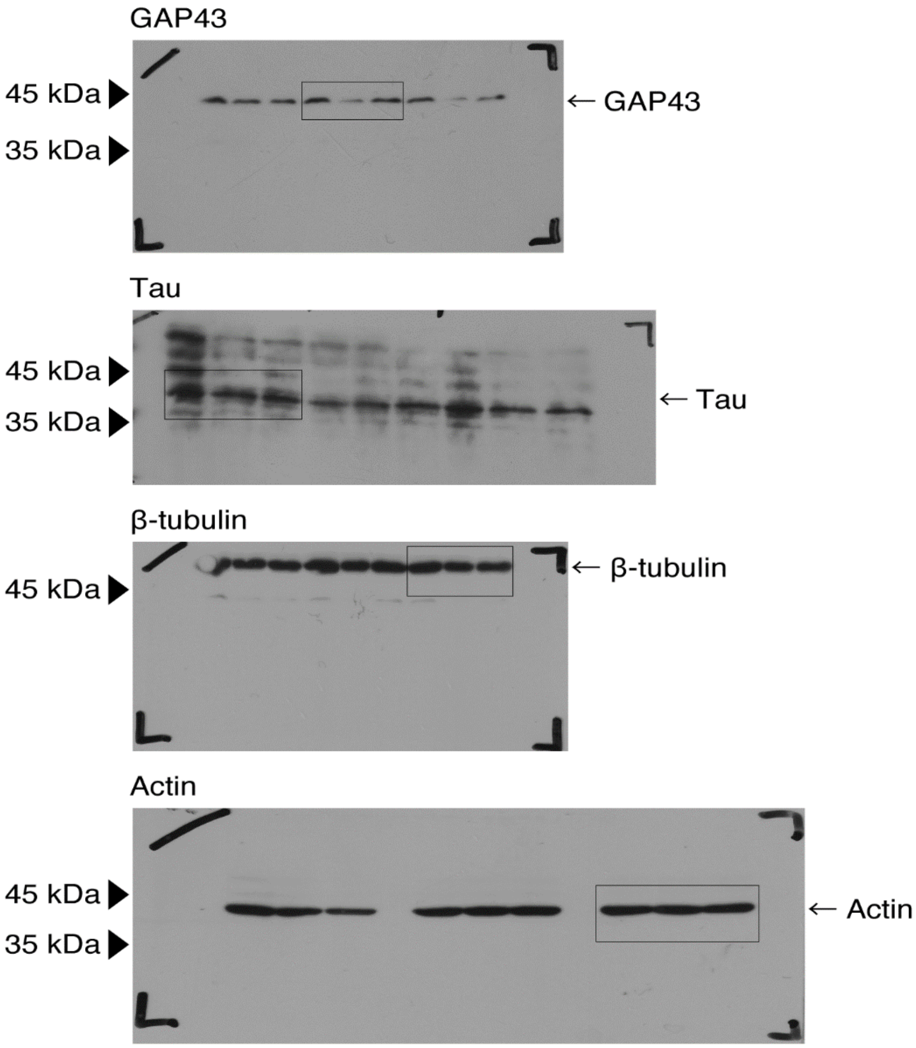

Figure S11. Original size gels saved as Figure 1 TIFF files.

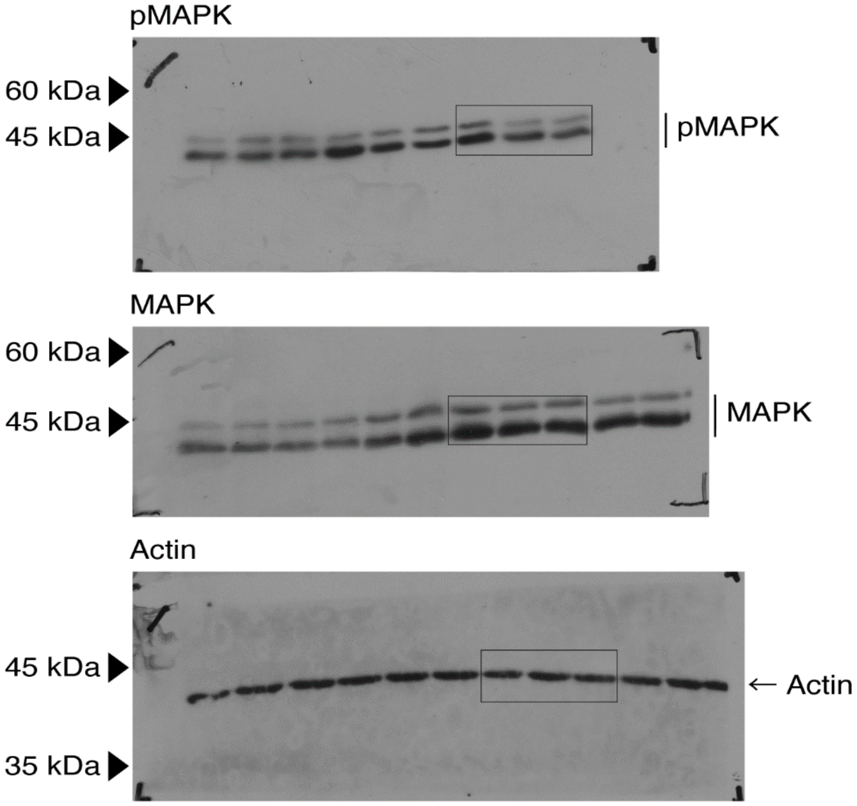

Figure S12. Original size gels saved as Figure 2TIFF files.

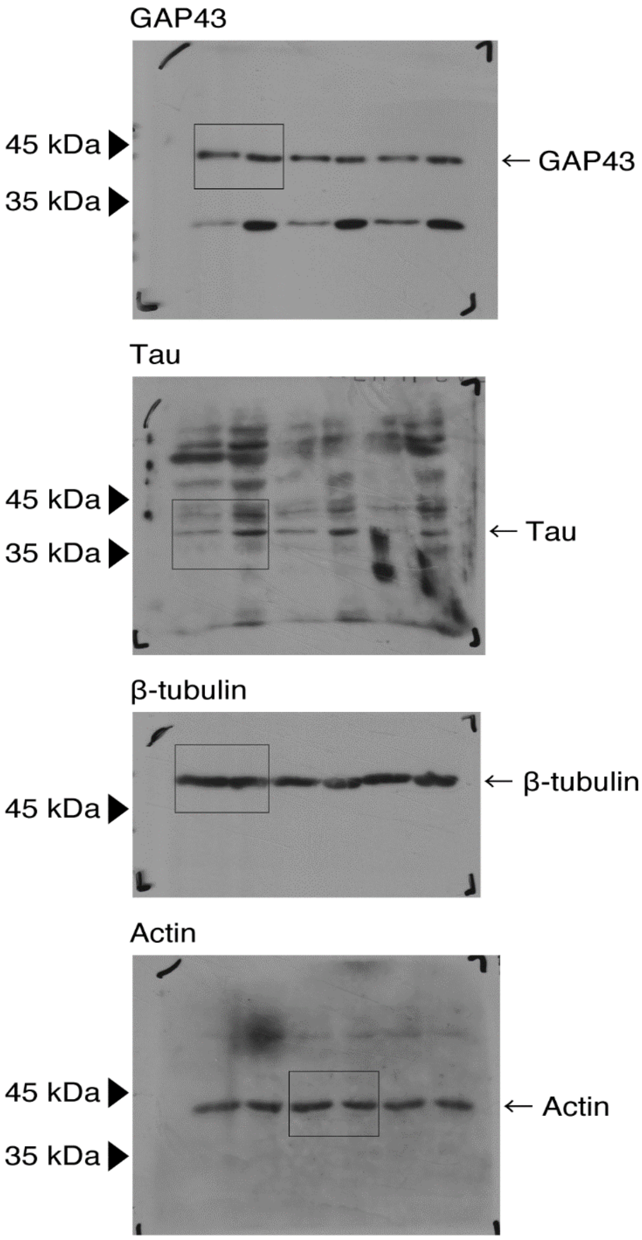

Figure S13. Original size gels saved as Figure 3 TIFF files.

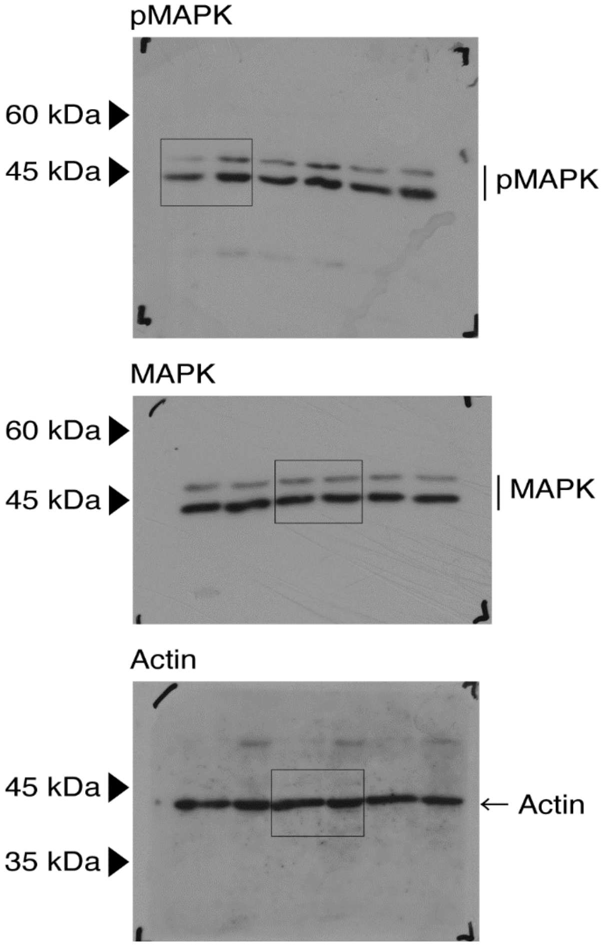

Figure S14. Original size gels saved as Figure 4 TIFF files.

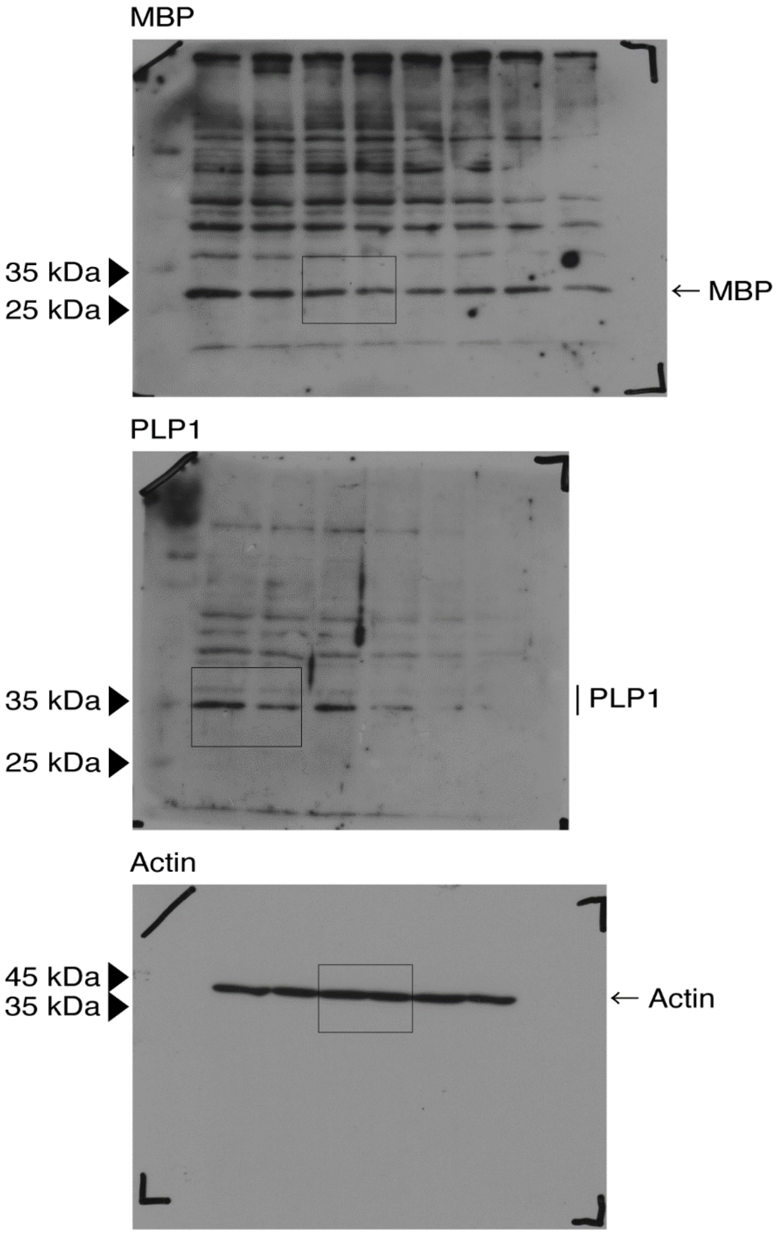

Figure S15. Original size gels saved as Figure 5 TIFF files.

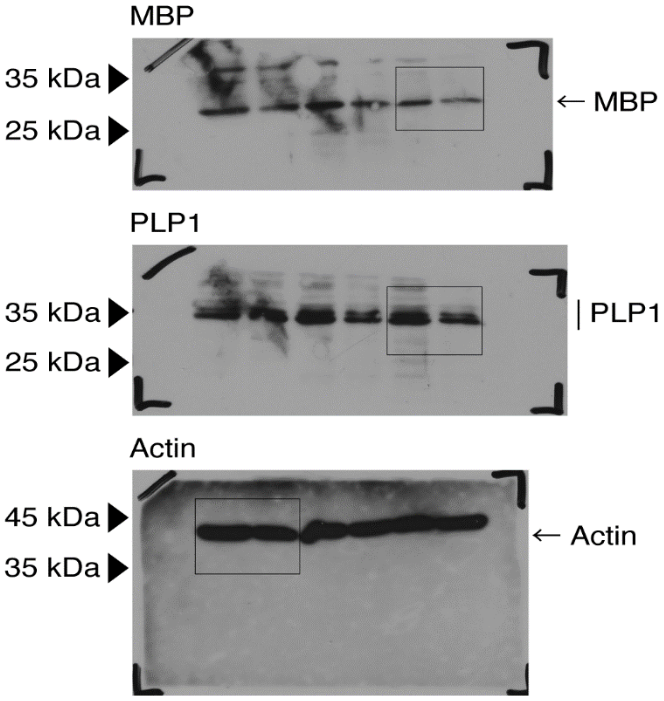

Figure S16. Original size gels saved as Figure 6 TIFF files.

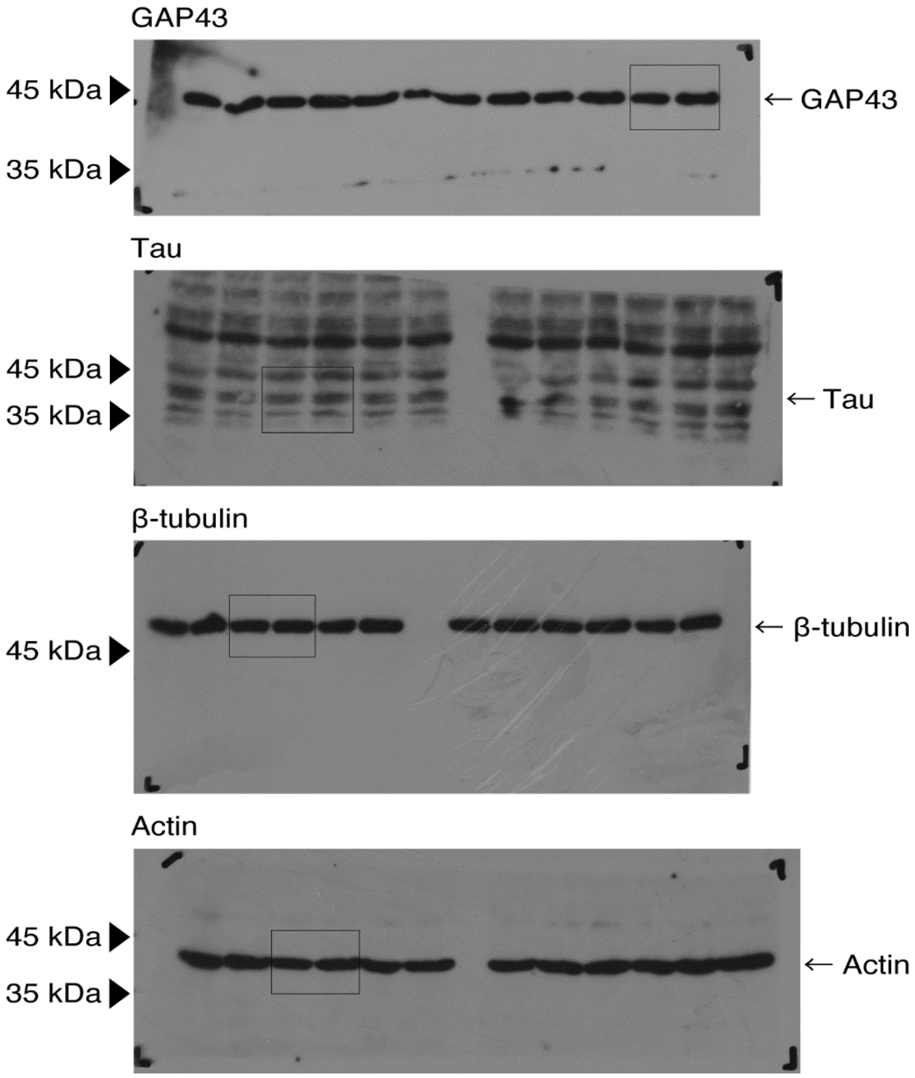

Figure S17. Original size gels saved as Figure S9 TIFF files.

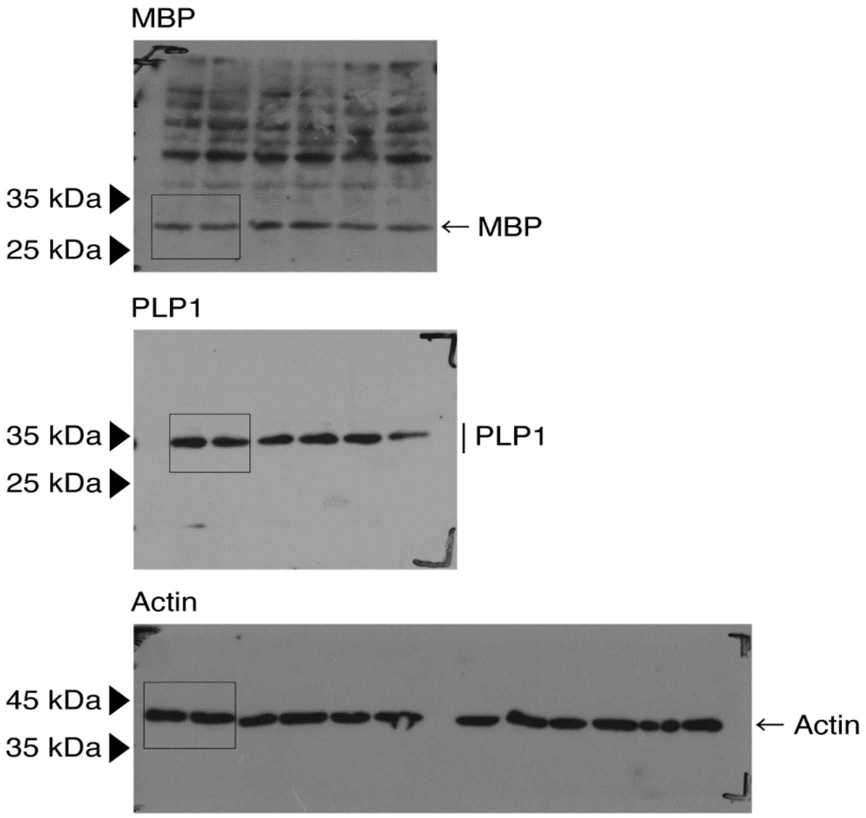

Figure S18. Original size gels saved as Figure S10 TIFF files.
